# Prevalence and pattern of retinopathy of prematurity at two national referral hospitals in Uganda: a cross-sectional study

**DOI:** 10.1186/s12886-023-03195-7

**Published:** 2023-11-22

**Authors:** Iddi Ndyabawe, Flavia Namiiro, Anita Tumwebaze Muhumuza, Jesca Nakibuka, Juliet Otiti, Anne Ampaire, Moses Kasadhakawo, Fransisco Msonge, Siyad Mohamed, Mary Nyanzi, Victor Spector Tumukunde, Andrew Weil Semulimi, David Mukunya, Dan Bwonya, Primrose Magala, Clare Gilbert, Nancy Maria Douat Dietrich, Patricia Zanotelli Cagliari, Anna Hedstrom, Mike Blair, Becca Jones, James Nyonyintono, Aisha Muhamad Doka, Bariirah Bushirah Nakitende, Hamish R. Graham, Susan Mary Carden, Rami Subhi, Grace Ssali Nsibirwa

**Affiliations:** 1 Department of Ophthalmology, School of Medicine, College of Health Sciences, Makerere University, Kampala, Uganda; 2 Department of Paediatrics and Child Health, Mulago Specialized Women and Neonatal Hospital, Kampala, Uganda; 3 Department of Ophthalmology, University of Antwerp, Antwerp, Belgium; 4 Department of Ophthalmology, Mulago National Referral Hospital, Kampala, Uganda; 5 Department of Paediatrics and Child Health, Kawempe National Referral Hospital, Kampala, Uganda; 6 Lung Institute, Department of Medicine, School of Medicine, College of Health Sciences, Makerere University, Kampala, Uganda; 7 Department of Community and Public Health, Faculty of Health Sciences, Busitema University, Tororo, Uganda; 8 Department of Ophthalmology, Mengo Hospital, Kampala, Uganda; 9 Moorfields Eye Hospital, London, UK; 10 Department of Clinical Research, London School of Hygiene & Tropical Medicine, London, UK; 11 Instituto de Oftalmologia de Joinville, Paulo, Brazil; 12 Department of Ophthalmology, School of Medicine, University of the Region of Joinville, Paulo, Brazil; 13 Department of Neonatology, University of Washington, Seattle, United States of America; 14 Department of Ophthalmology, Pritzker School of Medicine, University of Chicago, Hyde Park, United States of America; 15 Department of Paediatrics, Kiwoko Hospital, Luwero, Uganda; 16 Department of Special Needs Education, Entebbe Parents Senior Secondary School, Entebbe, Uganda; 17 School of Medicine, College of Health Sciences, Makerere University, Kampala, Uganda; 18 Department of Pediatrics, Faculty of Medicine, University of Melbourne, Melbourne, Victoria Australia

**Keywords:** Retinopathy of prematurity, Gestational age, Birth weight, Breast milk, Oxygen therapy, Sub-Saharan Africa, Uganda

## Abstract

**Background:**

Retinopathy of prematurity (ROP) is a leading cause of blindness in children and an ROP epidemic is predicted this decade in sub-Saharan Africa. With the increasing survival rate of preterm babies in Uganda, and no data on ROP prevalence, there is a need to assess the burden of ROP to inform preventive strategies and targeted screening.

**Methods:**

We conducted a two-center cross-sectional study of preterm (< 37 weeks gestational age) infants from the neonatal units of Kawempe National Referral Hospital (KNRH) and Mulago Specialised Women and Neonatal Hospital (MSWNH) from August 2022 to October 2022. An ophthalmologist examined all participants using an indirect ophthalmoscope with a + 20D convex lens and captured digital images using a Volk iNview™ Fundus Camera. The collected data were entered into Epidata 4.2 and exported to Stata 14.0 for analysis.

**Results:**

331 preterm infants enrolled in this study. The oxygen received was unblended. The mean gestational age was 30.4 ± 2.7 weeks, and the mean birth weight was 1597 ± 509 g. 18/101 (17.8%) were found to have any ROP amongst the preterm infants recruited from MSWNH, 1/230 (0.4%) from KNRH [95% CI] had any stage of ROP (i.e. stage 5). Of these, 8 (42.1%) had stage 2 ROP. Infants with a birth weight below 1500 g were 10 times more likely to have ROP than those among infants with a birth weight more than 1500 g [AOR: 10.07 (2.71–37.44)]. Infants who were not fed exclusively on breast milk had higher odds of having ROP than those exclusively fed on breast milk [AOR: 7.82(1.92–31.82)].

**Conclusion:**

6% of preterm infants born in two tertiary hospitals in Uganda were found to have ROP. Lack of exclusive feeding on breast milk and birth weight of less than 1500 g were strong predictors of ROP. The higher prevalence of ROP in MSWNH calls for cautious use of oxygen among preterms. We recommend targeted ROP screening for those at risk.

**Supplementary Information:**

The online version contains supplementary material available at 10.1186/s12886-023-03195-7.

## Background

More infants born preterm are surviving and thriving than ever before. Some preterm infants will have developmental disability, including visual complications [1, 2]. Retinopathy of prematurity (ROP) involves the progressive abnormal growth of the retinal blood vessels, that can lead to vision impairment or blindness [3]. Globally, approximately 184,700 preterm infants developed ROP in 2010 and more than 50,000 of these infants went on to get vision threatening ROP [4]. It is one of the leading cause of visual impairment and blindness among infants in high-income countries accounting for at least 50,000 cases of childhood blindness annually [1, 5]. For instance, 62%, 13% and 3% of cases of childhood blindness was due to retinopathy of prematurity in Mexico, the United States of America and United Kingdom respectively [6, 7]. The prevalence of retinopathy of prematurity is increasing rapidly with an “ROP epidemic” in sub-Saharan Africa predicted this decade (2015–2025). Infact 41.7% of preterm infants in Kenya were found to have ROP between 2010 and 2015 [6, 8–10].

The rising incidence of ROP in developing countries is largely attributable to the improvement of neonatal care and the subsequent improvement in survival of preterm infants as well as increased exposure to potentially modifiable risk factors. [11]. Previous studies have identified birth weight of less than 1.5 kg, gestational age less than 32 weeks, hyperoxia, sepsis, blood transfusion, respiratory distress, multiple pregnancies, and mechanical ventilation as predisposing factors for retinopathy of prematurity and its complications [9, 12–14].

The World Health Organization (WHO) recommends that all babies at risk of retinopathy of prematurity have a fundus examination by a trained observer 4–7 weeks after birth [11]. This recommendation is aimed at preventing blindness and increasing early detection and prompt treatment. However, implementation of this strategy is hindered by low number of neonatal intensive care units that have established ROP screening programs in Low and Middle Income Countries (LMIC) settings such as Uganda. The current capacity in Uganda for ROP screening is still very low. At this time, there are no national guidelines or previously published studies on ROP specific to Uganda. Furthermore, the national referral hospitals in this study are two of the largest public hospitals managing preterm infants, yet there was no formal ROP screening prior to initiation of a screening program by the primary investigator of this study (IN).

The challenges are lack of awareness amongst health workers, parents and health policy makers as regards to ROP, its detrimental effects to a child, the need for a regular nationwide ROP screening program and the inevitable need to offer affordable treatment for ROP to those preterm babies found to have vision-threatening ROP [8].

Uganda has a preterm birth rate of 13.6 births per 1000 live births yearly but no data on ROP prevalence and no formal ROP screening program [15]. Therefore, this study aimed to assess the prevalence, patterns (stages) and associated factors of ROP in the two largest public hospitals (KNRH and MSWNH) receiving preterm infants in Kampala, Uganda. Results from this study would guide the development of policies regarding ROP prevention and potential development of a screening and treatment program in Uganda.

## Methods

### Study design

We conducted a two-center cross-sectional prevalence study, from August to October 2022.

### Study setting and population

This study was conducted at the Neonatal Units of Kawempe National Referral Hospital (KNRH) and Mulago Specialised Women and Neonatal Hospital (MSWNH), located in the Kawempe division in Uganda’s capital, Kampala. Both hospitals serve as the teaching hospitals of Makerere University College of Health Sciences. KNRH offers free health services to patients while MSWNH offers free and private health services. During the study period, the noted cost for admission of a preterm infant at MSWNH was about 150,000 Uganda Shillings per night (**≈** 40 US Dollars).

The Neonatal Intensive Care Unit (NICU) ward of KNRH admits about 20 neonates daily, with 80–100 preterm babies cared for at a given time in the NICU. A minority (about ten or fewer) stay on the ward for over four weeks, but the numbers vary. The neonatal follow-up clinic of KNRH is integrated within the pediatric Acute Care Unit) clinic, reviewing approximately 100 preterm babies weekly. Around 80 of these preterm infants are four or more weeks old.

MSWNH is a component of Mulago National Referral Hospital (MNRH), the largest hospital in Uganda. The NICU ward of MSWNH admits about five neonates per day, with 50–60 preterm babies cared for at a given time in this NICU. About five or fewer stay on the ward for four weeks or longer, but the numbers also vary. The neonatal follow-up clinic of MSWNH reviews approximately 30 neonates a week. Around 15 of these preterm infants are four weeks old and older. MSWNH receives preterm babies referred in from KNRH, other health facilities around Kampala, and a few from different regions of Uganda.

The NICU of MSWNH is well equipped with more advanced neonatal care services than that of KNRH. For example, preterm babies at KNRH receive basic unblended oxygen therapy on a ‘shared-basis’ via improvised ‘Y-connectors’. The babies at MSWNH have ready supply of unblended oxygen for low-flow and continuous positive airway pressure (CPAP), with every bed having direct connection to the central oxygen supply pipes embedded within the walls and each preterm infant having its own oxygen saturation monitor 24/7. Anecdotal reports indicate that KNRH refers most very preterm infants, especially those with birthweight below 1000 grams and other neonatal risks, to MSWNH for further management.

NICUs of both facilities have an appended space for Kangaroo Care for stable preterm infants and admit to the NICU if they have or are at risk of complications or danger after birth. The neonatal follow-up clinic receives preterm babies discharged from the NICU, reviewing regularly until they have attained 2.5 kg, then discharging to the preterm/LBW clinic for subsequent review until one year of age. Upon discharge from the NICU, the patient’s data is summarized on a discharge form carried by the mother when she comes to the outpatient neonatal follow-up clinic. The preterm infants who were discharged early were followed-up via the Preterm Review clinics and ROP screening done for those found to fit in the inclusion criteria during the study duration, irrespective of birth weight.

In these facilities, the neonates are reviewed and managed by qualified paediatricians, medical officers and trained neonatal nurses.

### Study population and eligibility criteria

#### Study population

Preterm babies managed at KNRH and MSWNH during the study period.

### Inclusion criteria

All pre-term neonates who were admitted to the neonatal wards, and those who attended the neonatal follow-up clinics during the study period with this description:

1. Born less than 37 weeks gestational age with post-menstrual age (PMA) < 49 weeks.
2. if GA > 30 weeks, neonates who were at least four weeks old.
3. if GA < 30 weeks, neonates who were at least 4–9 weeks old (as long as the PMA reaches a minimum of 31 weeks).
4. Those whose parents/ guardians consented to enrolment in the study.

The gestational age was assessed by reviewing the Ballard score recorded in the patient’s file or the last normal menstrual cycle data reported by the mother, or by ultrasound scan measurements done within the first trimester of pregnancy.

**Table:** 

Recommended Timing of First Exam Based on Gestational Age in the United States		
Gestational Age at Birth	Postmenstrual age (PMA) (weeks)	Chronologic (weeks)
22 weeks	31	9, consider earlier screening per clinical judgment
23 weeks	31	8, consider earlier screening per clinical judgment
24 weeks	31	7
25 weeks	31	6
26 weeks	31	5
27 weeks	31	4
28 weeks	32	4
29 weeks	33	4
30 weeks	34	4
> 30 weeks with high-risk factors		4

(Fierson WM et al., 2019)

### Exclusion criteria

1. Very sick neonates deemed too unstable for eye examination by neonatal clinical staff (5 babies).
2. Very hazy corneas that could not be seen through using an indirect ophthalmoscope and the Volk iNview Fundus Camera (2 babies. These were referred to the vitreo-retinal specialist for further assessment).

### Sample size and sampling procedure

Sample size calculation for the prevalence was determined using the Kish Leslie formula at 95% confidence interval and error within 5% of one proportion for the prevalence (ref). Sample size calculation for factors associated with ROP was done using the difference in two independent proportions formula by Fleiss (ref). Assuming 50% of preterm infants undergoing treatment in neonatal care units were at risk of developing ROP whilst basing on estimates by Onyango et al., 2018 (ref), the minimum sample size for this study was calculated as 385 participants [9]. To answer both objectives of prevalence and associated factors of ROP in KNRH and MSWNH, a sample size of 331 was used. The participants were enrolled consecutively from the two hospitals, with 101 neonates from MSWNH and 230 neonates from KNRH.

### Sample size calculation for factors associated with retinopathy of prematurity (ROP)

We used the sample size for two proportions method (The Fleiss method);

$$\varvec{n}=\frac{{{[\varvec{Z}}_{\varvec{\alpha }/2}\sqrt{\varvec{p}(1-\varvec{p})(\raisebox{1ex}{$1$}\!\left/ \!\raisebox{-1ex}{${\varvec{q}}_{1}$}\right.+\raisebox{1ex}{$1$}\!\left/ \!\raisebox{-1ex}{${\varvec{q}}_{2}$}\right.)}+{\varvec{Z}}_{\varvec{\beta }}\sqrt{{\varvec{p}}_{1}\left(1-{\varvec{p}}_{1}\right)\raisebox{1ex}{$1$}\!\left/ \!\raisebox{-1ex}{${\varvec{q}}_{1}$}\right.+{\varvec{p}}_{2}(1-{\varvec{p}}_{2})\raisebox{1ex}{$1$}\!\left/ \!\raisebox{-1ex}{${\varvec{q}}_{2}$}\right.}]^{2}}}{{({\varvec{p}}_{1}-{\varvec{p}}_{2})}^{2}}$$

**Where;**

Z_α/2_ is the standard normal value corresponding to the level of significance (e.g. for a confidence level of 95%, α is 0.05, and the critical value is 1.96),

Z_β_ is the standard normal value corresponding to the power of the study (e.g. for a power of 80%, β is 0.2, and the critical value is 0.84),

Taking the ratio of group Two: group One = 1 and assuming no difference in all groups and a two-sided test. p = p_1_q_1_ + p_2_q_2_.

Using the study by (Hakeem et al., 2019), the prevalence of ROP was found to be 19.2%.

Using gestational age as the main associated factor, the proportion in group one was.

**p**_**1**_ **= 0.148, and the proportion in group two was p**_**2**_ **= 0.45.**

Based on the above formula, the calculated sample size was a minimum of 150 participants to answer objective 2. Given that the larger of the two sample sizes is 298, the final sample size was 298 preterm neonates.

Considering a correction of 10% non-response, we adjusted using the formula 298/(100 − 10)% and arrived at a sample size of 331 preterm neonates to be screened during the study.

So, to answer both objectives of prevalence and associated factors of ROP in KNRH and MSWNH, a sample size of 331 was used.

### Data collection

An ophthalmology trainee (NI) trained in ROP examinations conducted all participant screening, interviewing and eye examinations. They used interviewer-administered questionnaires to obtain information on the neonatal and maternal characteristics and used a consultation form to record retinopathy of prematurity from the indirect ophthalmoscopy and fundus camera results. All examinations included capture of retinal images for quality checking by a senior ophthalmologist who is experienced in ROP examination, and discussion where diagnostic classification was unclear.

### Data analysis

Data were coded and entered into electronic Epi Data Version 4.2 and then exported to Stata Version 14 for cleaning and analysis. The main outcome variable for the study was ROP whose severity was categorized according to the stage of ROP in the respective zones of the retina. For baseline characteristics, the Pearson’s Chi- squared test was used to assess for differences in proportions between the two hospitals. Univariate and multivariate logistic regression analyses were used to assess the association between the demographic, clinical, treatment factors and retinopathy of prematurity. For multivariable analysis, factors that had a p-value of < 0.2 at bivariate analysis were considered in building a model using logistic regression analysis.

### Ethical considerations

We obtained ethical approval from the Department of Ophthalmology Makerere University and the School of Medicine Research and Ethics Committee (SOMREC) of Makerere University (Mak-SOMREC-2022-313). All parents of the participants were informed of the intentions of the research and only those who accepted to sign informed consent form were involved and given the liberty to withdraw from the study as and when they wish to.

## Results

### Population characteristics

Out of 331 preterm infants enrolled, half (52.3%) were female (Table 1). The mean gestational age was 30.4 ± 2.7 SD weeks and the mean birth weight was 1597 g ± 509 SD. The populations of the preterm neonates seen at the different facilities were different (Table 2). Neonates from MSWNH had a significantly lower gestation age (median 29 vs. 31weeks: p = 0.007) and birth weight (median 1170 g vs. 1700 g: p < 0.001) compared to those from KNRH.

**Table 1:** Demographic characteristics of the participants

	Mulago Specialised Women and Neonatal hospital		Kawempe National Referral Hospital		Total	
	Frequency	%	Frequency	%	Frequency	%
**Sex of baby**						
Male	46	45.5	112	48.7	158	47.7
Female	55	54.5	118	51.3	173	52.3
Total	101	100	230	100	331	100
**Mothers education level**						
No education	0	0	3	1.6	3	1.1
Primary level	9	10.2	38	20	47	16.9
Secondary level	31	35.2	119	62.6	150	54
Tertiary level	48	54.5	30	15.8	78	28.1
Total	88	100	190	100	278	100
**Birth weight**						
Less than 1500 g	76	75.2	56	24.3	132	39.9
1500 g and more	25	24.8	174	75.7	199	60.1
Total	101	100	230	100	331	100
**Gestational age**						
Less than 32 weeks	80	79.2	115	50	195	58.9
More than 32 weeks	21	20.8	115	50	136	41.1
Total	101	100	230	100	331	100
**Chronological age**						
0 to 5 weeks	45	44.6	102	44.3	147	44.4
More than 5 weeks	56	55.4	128	55.7	184	55.6
Total	101	100	230	100	331	100

**Table 2:** Comparison of preterm neonates from the two hospitals

	MSWNH (N = 101)	KNRH (N = 230)	p-value for difference in proportions
**Gestational age** (median, IQR)	29 (28–31)	31 (28–32)	0.007
**Birthweight** (median, IQR)	1170 (900–1490)	1700 (1500–2000)	< 0.001
**Chronological age** (median days, IQR)	5 (4–8)	5 (4–8)	0.929
**IVH**	5/95 (5.3%)	1/228 (0.43%)	0.0034
**PDA**	1/95	0	
**Sepsis**	42/94 (44.7%)	62/222 (27.9%)	0.0038
**RDS**	85/98 (86.7%)	136/218 (62.4%)	< 0.0001
**O2 therapy**	99/101 (98.1%)	157/227 (69.2%)	< 0.0001
**Duration of O2 therapy** (median days, interquartile range)	7 (4–14), n = 98	3 (2–7), n = 155	< 0.0001 (* difference in medians)
**Blood transfusion**	24/100 (24%)	6/227 (2.6%)	< 0.0001
**Phototherapy**	62/99 (62.6%)	51/228 (22.4%)	< 0.0001
**APGAR Score** (median, IQR)	8 (8–9)	9 (9–10)	< 0.0001
**Haemoglobin** (median)	14 (11–17) n = 62	10.5 (9–14) n = 6	

Key: *IVH* Intraventricular Hemorrhage, *PDA* Patent Ductus Arteriosus, *RDS* Respiratory Distress Syndrome

### Prevalence and patterns of ROP

ROP was found in 18/101 (17.8% [11.5–26.6]) infants in MSWNH and1/230 (0.4% [0.1–3.1]) infants from KNRH.

**Fig. 1:**
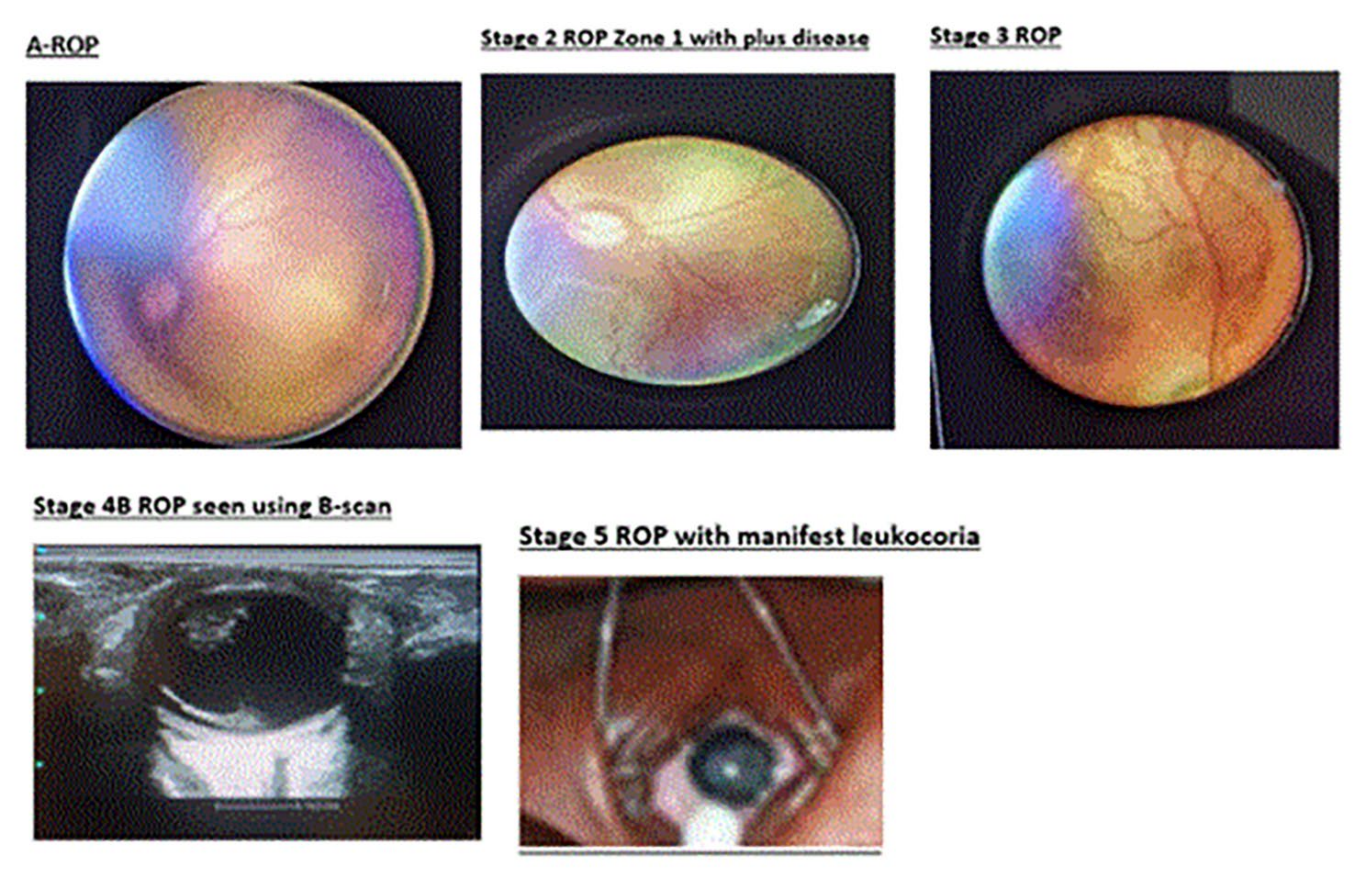
Photographs of different stages of retinopathy of prematurity seen during this study. *Note: Informed consent was obtained from the parents of the respective infants to publish the image of neonates in “*Figure 1*”*

Among those with ROP, 7(36.8%) had Stage 2 Zone II in either the left or the right eye. One neonate examined at KNRH was found with Stage 5 ROP (i.e. end-stage ROP) in both eyes and no other infant was seen to have ROP in KNRH. Of the preterm neonates with ROP, 2(10.5%) presented with stage 1, 8(42.1%) with stage 2, 3(15.8%) with stage 3, 2(10.5%) with stage 4, 1(5.3%) with stage 5 and 3(15.3%) with A-ROP (Table 3; Fig. 1). All cases found to have ROP during this study had bilateral disease and similar staging of ROP, except for one patient. This is outlined well in Table 3 explaining the pattern of ROP.

Six (1.8%) had tortuous blood vessels, while one neonate (0.3%) had leukocoria.

**Table 3:** Patterns of ROP

Stage of ROP	Left Eye		Right Eye		TOTAL	
	Frequency	Percentage	Frequency	Percentage	Frequency	Percentage
Stage 1 Zone 2	3	15.8	2	10.5	2	10.5%
Stage 2 Zone 1			1	5.3		
Stage 2 Zone 2	7	36.8	7	36.8	8	42.1%
State 3 Zone 2	3	15.8	3	15.8	3	15.8%
Stage 4 A	1	5.3	1	5.3		
Stage 4 B	1	5.3	1	5.3	2	10.5%
Stage 5	1	5.3	1	5.3	1	5.3%
A-ROP	3	15.8	3	15.8	3	15.8%
Total	19	100	19	100	19	100

### Factors associated with retinopathy of prematurity (ROP)

On univariate analysis, the following factors were associated with ROP: gestational age, birth weight, intraventricular hemorrhage, patent ductus arteriosis, respiratory distress, oxygen therapy, history of phototherapy, APGARS, and anemia. After checking for collinearity, the following factors remained significant in the multivariate analysis; low birth weight, chronological age and type of feeding were significantly associated with retinopathy of prematurity (Table 4).

**Table 4:** Multivariable analysis for factors associated with ROP

	Crude OR (95% CI)	p-value	Adjusted OR (95% CI)	p-value
**Low birth weight**				
Less than 1500 g	9.01 (2.57–31.59)	0.001	10.07 (2.71–37.44)	0.001**
1500 g or more	1		1	
**Type of Feed**				
Mother’s Breast Milk	1		1	
Other (Formula milk)	9.56(2.62–34.85)	0.001	7.82(1.92–31.82)	0.004**

*P < 0.05 ** p < 0.01 ***p < 0.001

## Discussion

We assessed the prevalence of ROP in 2 tertiary units in Uganda. Out of 331 preterm babies, 19 (5.7%) had ROP of any stage, with 18 of these cases from one hospital (i.e. MSWNH).

Our findings suggest that the burden of undiagnosed ROP in Uganda is significant. Point estimates of prevalence will depend on factors associated with context (e.g. access to quality antenatal, perinatal and postnatal care), quality and level of care (e.g. survival of preterm infants, and higher level therapies including mechanical ventilation), and study design (e.g. inclusion criteria, specifically gestation and birth weight). Previous studies from comparable settings in Botswana and Rwanda have reported similar prevalence of 7.3–11% when screening infants < 34 weeks and < 35 weeks. [16]

The difference in prevalence between the two hospitals is multifactorial. MSWNH offers free and privatized services, is a training centre for neonatology, and is a referral centre that receives infants from surrounding facilities including KNRH. This may reflect the higher risk patients that survive at MSWNH vs. KNRH. The gestational age and birthweight of infants screened in MSWNH was therefore lower than KNRH, with a higher proportion with sepsis, RDS, and blood transfusions. One postulation would be the smaller KNRH patients may not survive long enough to get ROP. Overall, the difference in prevalence likely reflects baseline differences in the patient populations. Data points from both sites are definitely important. Oxygen therapy contributes to ROP by affecting retinal vascularization through regulating vascular endothelial growth factor [17]. In line with this, a greater proportion of infants in MSWNH received oxygen (98% vs 69%), for a longer duration (7 days vs 3 days). Each of the babies at MSWNH had its own oxygen saturation monitor which was working 24/7. Difference in oxygen exposure between the two sites is very remarkable: In MSWNH, the noted use of oxygen via ventilator or CPAP would be “true” 100% oxygen exposure, vs. the split oxygen via nasal cannula called Y-connectors in KNRH which could be below 50% oxygen. In both facilities, all babies who were on oxygen received unblended oxygen.

All the babies with ROP identified in this study will need ongoing follow-up. We found stage 2 ROP in 42% of those with ROP. At least 13 (68.4%) were treatment-requiring ROP. The current cost of an intravitreal injection for avastin (bevacizumab) is 600,000/= (about $ 158). Families would also have to pay for travel and accommodation costs. Treatment is currently only offered in very few facilities in Kampala, Uganda’s capital which include Mengo Hospital, Nsambya Hospital and Dr. Agarwal’s Eye Hospital. By the time of doing this study, and even currently in Uganda, laser therapy is unavailable for treatment of ROP. Laser therapy, the gold standard for ROP treatment, is surely a possible treatment of ROP in preterm infants with large birth weight and mature Gestational Age. Besides, such a treatment might decrease the follow-up burden for the families. Lack of access to this important treatment option remains a problem throughout sub-Saharan Africa [18].

Three (15.8%) babies with ROP had advanced ROP (Stage IV and V), that even with treatment has a poor outcome of only minimal ambulatory vision. The preterm baby who had Stage 5 ROP was the only one found to have ROP amongst all the 230 screened at KNRH during the study period. This child was rendered blind as per treatment capabilities in Uganda. The surgical techniques needed for Stage 5 ROP are unique and very demanding, with successful results after surgery seen in about 20–50% of the cases, however, this is currently not available in Uganda [19, 20]. Such preterm babies are referred to Aga Khan University Hospital in Nairobi, Kenya where the surgery cost about $7,000 for each baby.

Our data have reproduced the higher risk of ROP in infants with low birth weight (independent of gestation), consistent with an Ethiopian study on infants attending an eye clinic in Minilk-11 hospital found that infants born with a very low birth weight below 1000 g or between 1000 and 1500 g were 39 times and 12 respectively more likely to develop ROP compared to those with greater than 1500 g [21]. Our study has also demonstrated the protective role of exclusive breastfeeding which is in line with other studies and the protective role of breast milk is attributed to the long chain polyunsaturated fatty acids that counteract the oxygen free radicals hence protecting the baby from getting ROP [22]. Our suspicion is that the increased risk in patients who received “other” feeds besides mother’s milk may be related to other risks of those babies. Given the emphasis on maternal milk, it is a rarity for patients to get other feeds and may be a high-risk group (i.e. maternal death/abandonment/etc).

This is the very first study on the prevalence, pattern (stages) and associated factors of ROP among preterm infants in Uganda. It provides strong evidence of the undiagnosed burden of ROP in Uganda, and a strong basis for advocating for screening and treatment programs. The risk factors for ROP identify critical areas for intervention and prevention. In our study, these areas included the prevention of low-birth-weight, the promotion of exclusive breastfeeding, and interventions to improve the quality of care for preterm newborns, especially the safe and rational use of oxygen.

The current capacity in Uganda for ROP screening is still very low given the fact that this was the very first study of ROP burden in two of the largest public hospitals which are even national referral hospitals managing preterm infants in the country.

The challenges are lack of awareness amongst health workers, parents and health policy makers as regards to ROP, its detrimental effects to a child, the need for a regular Nation-wide ROP screening program and the inevitable need to offer affordable treatment for ROP to those preterm babies found to have treatment-requiring ROP.

Since ending the study, the principal investigator (NI) has continued to reinforce the ROP screening program he commenced in these two tertiary hospitals MSWNH and KNRH. The principal investigator (NI) also approached various NICUs both private and public as well as those in the urban and in the rural areas to inform them of the crucial need of initiating an ROP screening program in their centres and is willing to help them in this.

The authors of this paper recommend a collaboration between ophthalmology, neonatology, nursing, paediatrics and health policy makers so as to establish a robust nation-wide ROP screening program in Uganda, then subsequently draft the much-needed ROP national guidelines for Uganda.

### Limitations of the study

The large difference in prevalence between the two sites highlights the importance of context. Our findings are therefore not generalizable to all facilities caring for preterm newborns in Uganda, and our findings underline the importance of conducting larger prevalence surveys to inform the design of a screening program. Our study did not collect detailed information on risk factors (e.g. antenatal care, or aspects of neonatal quality of care), and this will be important in future studies to help inform interventions for prevention of ROP.

## Conclusion

The prevalence of ROP among infants born < 37 weeks gestational age in tertiary hospitals in Uganda is significant at 5.7%. Prevalence was significantly different between the two facilities with MSWNH having a higher prevalence than KNRH (17.8% Vs 0.4%). 7 infants (36.8%) had Stage 2 of ROP and 13 (68.4%) had treatment-requiring ROP The most significant risk factors for ROP were long duration of oxygen therapy, pretems not being exclusively fed on breast milk and low birth weight less than 1500 g.

### Electronic supplementary material

Below is the link to the electronic supplementary material.

Supplementary Material 1

Supplementary Material 2

Supplementary Material 3

Supplementary Material 4

Supplementary Material 5

Supplementary Material 6

Supplementary Material 7

## Data Availability

The dataset used and analyzed during this study is available from the corresponding author upon request.

## Abbreviations

ROP: Retinopathy of prematurity
KNRH: Kawempe National Referral Hospital
MSWNH: Mulago Specialized Women and Neonatal Hospital
