# Supplementary figures and images for: Prevalence and pattern of retinopathy of prematurity at two national referral hospitals in Uganda: a cross-sectional study
Source: BMC Ophthalmol. 2023 Nov 22;23:478. doi: 10.1186/s12886-023-03195-7 (PMC10664491; doi:10.1186/s12886-023-03195-7)

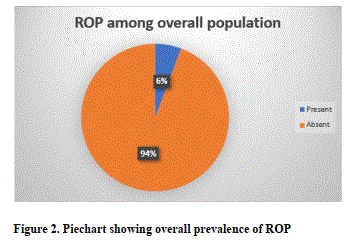

Supplement: Supplementary file 1 — Supplementary Material 1 [file 12886_2023_3195_MOESM1_ESM.png]

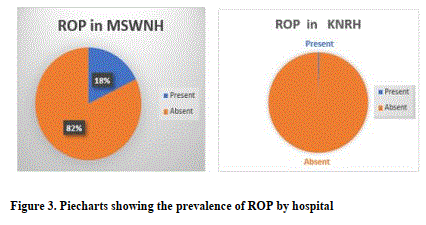

Supplement: Supplementary file 2 — Supplementary Material 2 [file 12886_2023_3195_MOESM2_ESM.png]

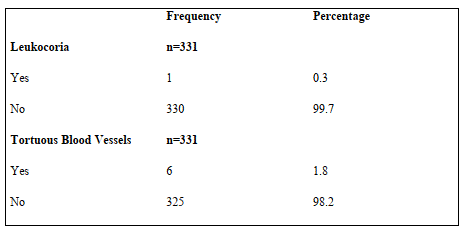

Supplement: Supplementary file 3 — Supplementary Material 3 [file 12886_2023_3195_MOESM3_ESM.png]

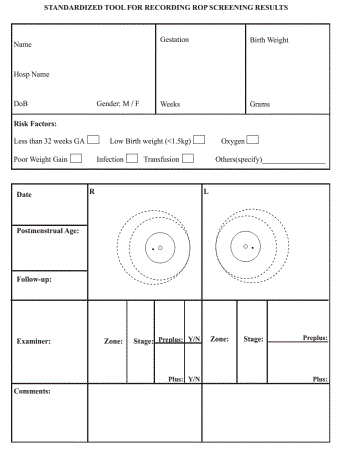

Supplement: Supplementary file 4 — Supplementary Material 4 [file 12886_2023_3195_MOESM4_ESM.png]

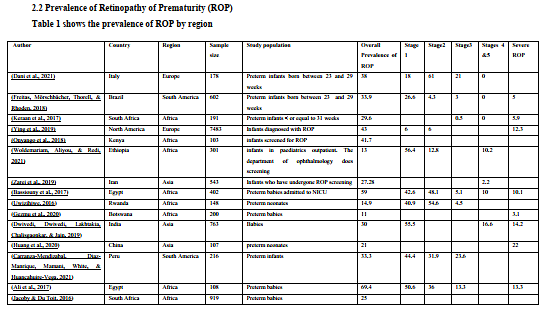

Supplement: Supplementary file 5 — Supplementary Material 5 [file 12886_2023_3195_MOESM5_ESM.png]

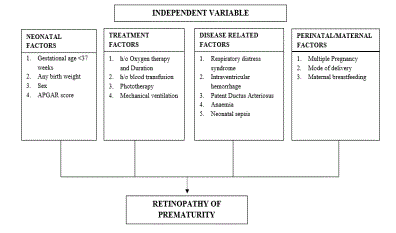

Supplement: Supplementary file 6 — Supplementary Material 6 [file 12886_2023_3195_MOESM6_ESM.png]

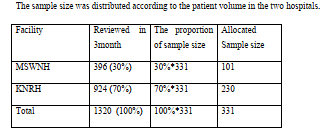

Supplement: Supplementary file 7 — Supplementary Material 7 [file 12886_2023_3195_MOESM7_ESM.png]
